# Supplementary material for: Identification and Validation of Reference Genes for Gene Expression Analysis in Schima superba
Source: Genes (Basel). 2021 May 13;12(5):732. doi: 10.3390/genes12050732 (PMC8153319; doi:10.3390/genes12050732)
Supplement: Supplementary file 1 [file genes-12-00732-s001.zip › Additional file/Additional file 1 Figure S1.docx]

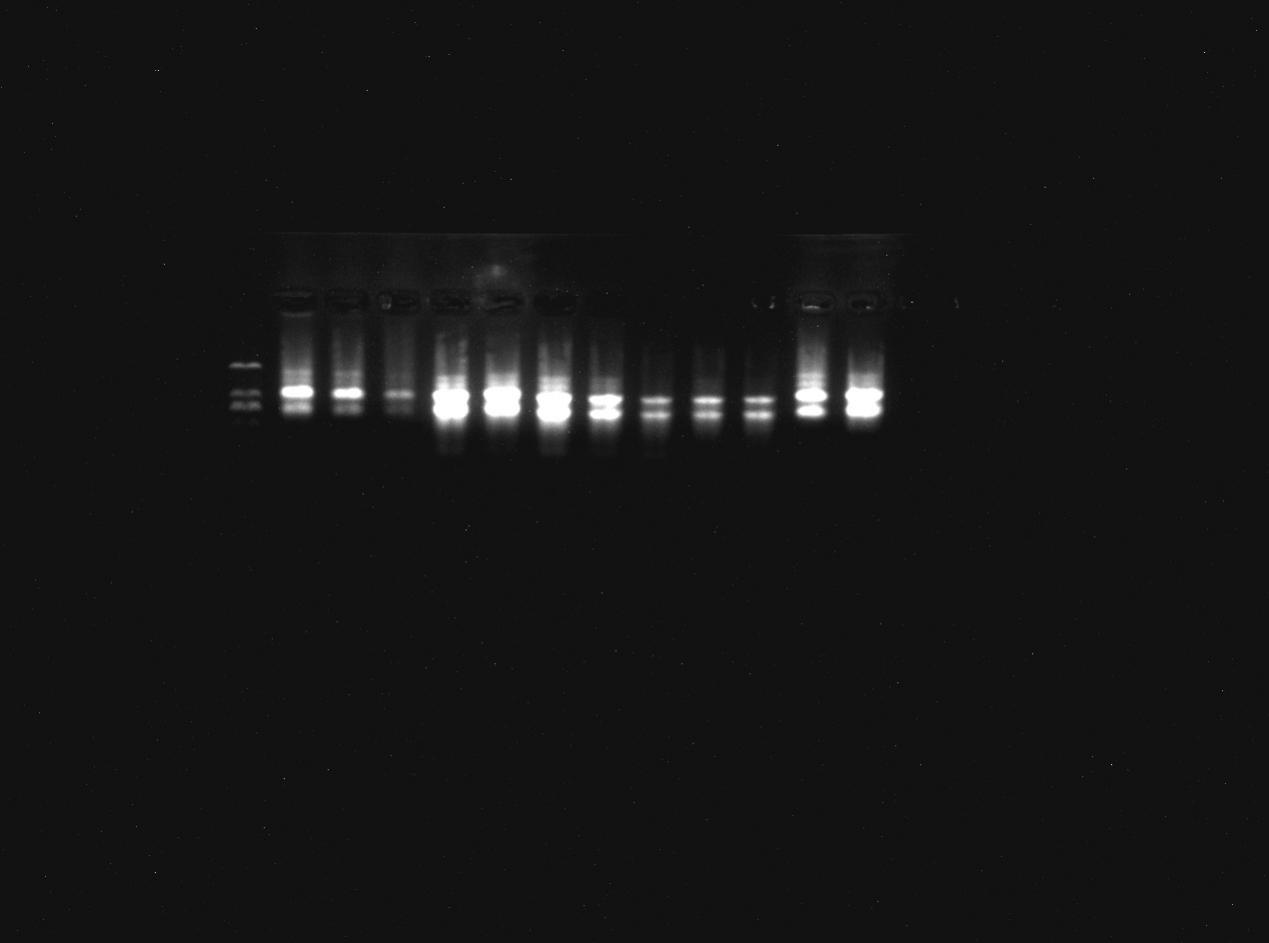


Figure S1-A Agarose gel electrophoresis analysis of total RNA


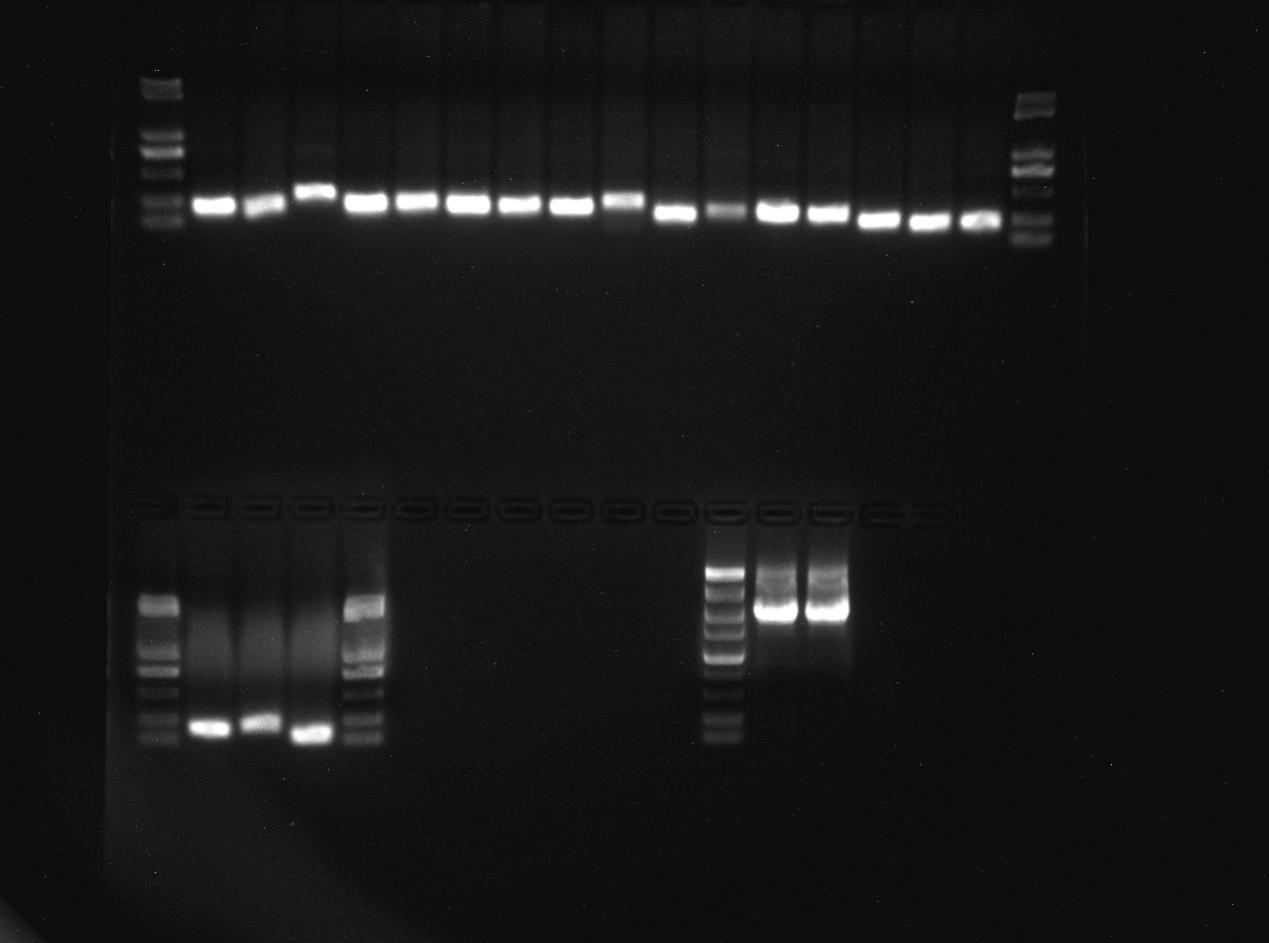


M 1 2 3 4 5 6 7 8 9 10 11 12 13 14 15 16 M

M 17 18 19 M

750

250

750

250

Figure S1-B Agarose gel electrophoresis analysis of candidate gene PCR products

M：Marker 2K；1-19：*SsuACT*、*SsuHis*、*SsuTUA1*、*SsuTUA2*、*SsuUBC1*、*SsuUBC2*、*SsuUBCJ2*、*SsuUBC17*、*SsuMDH*、*SsueIF5*、*SsuGTP*、*SsuCal7*、*SsuRIB*、*SsuMet2*、*ColGAPDH*、*SsuTUB*、*SsuCas*、*SsuUDP*、*SsuGAPDH*
