# Supplementary material for: Identification and Validation of Reference Genes for Gene Expression Analysis in Schima superba
Source: Genes (Basel). 2021 May 13;12(5):732. doi: 10.3390/genes12050732 (PMC8153319; doi:10.3390/genes12050732)
Supplement: Supplementary file 1 [file genes-12-00732-s001.zip › Additional file/Additional file 2 Figure S2.docx]

| 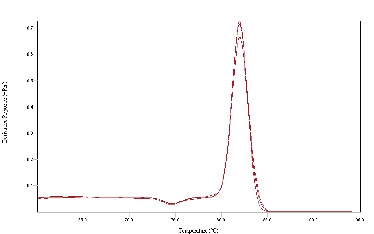 | 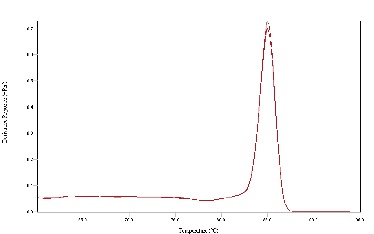 | 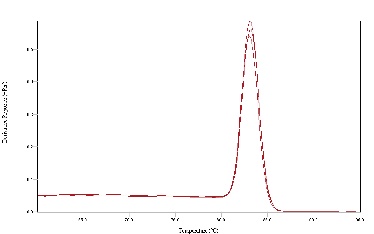 |
| --- | --- | --- |
| *CajGAPDH* | *SsuCal7* | *SsuCas* |
| 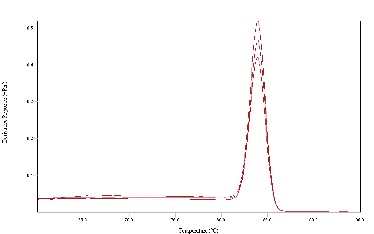 | 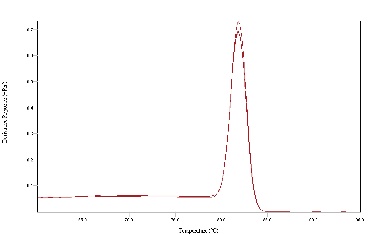 | 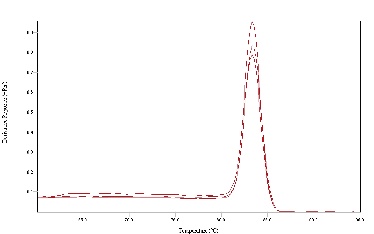 |
| *SsuACT* | *SsueIF5* | *SsuGAPDH* |
| 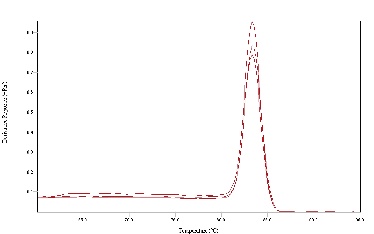 | 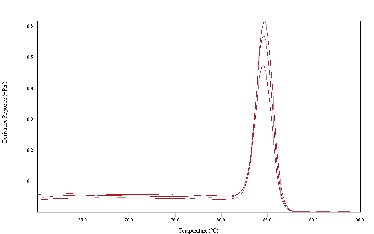 | 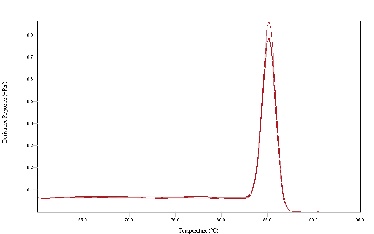 |
| *SsuGTP* | *SsuHis* | *SsuMDH* |
| 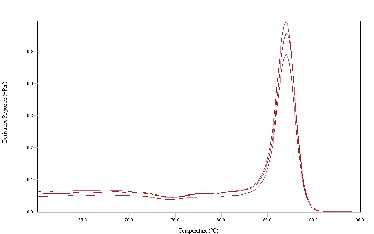 | 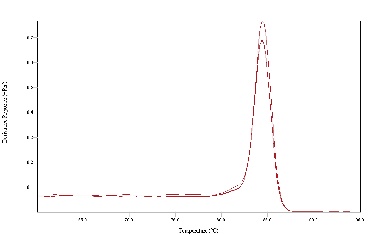 | 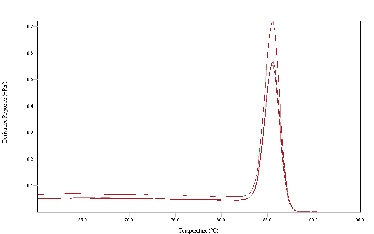 |
| *SsuMet2* | *SsuRIB* | *SsuTUA1* |
| 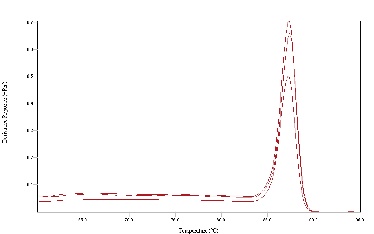 | 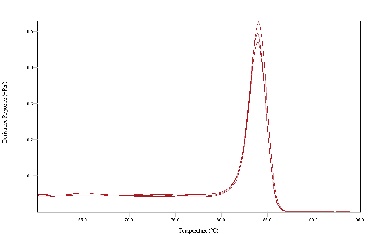 | 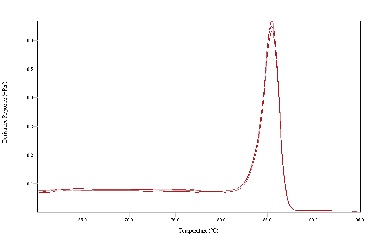 |
| *SsuTUA2* | *SsuTUB* | *SsuUBC1* |
| 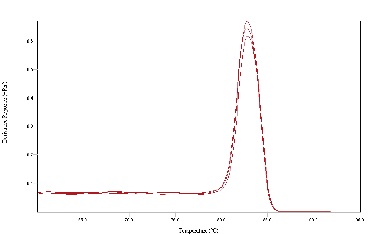 | 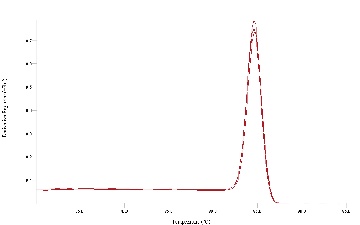 | 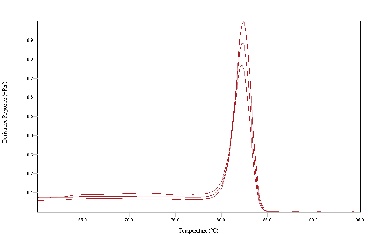 |
| *SsuUBC2* | *SsuUBC17* | *SsuUBCJ2* |
| 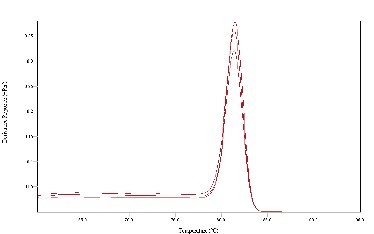 | 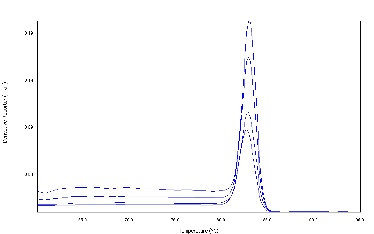 |  |
| *SsuUDP* | *SsuSND1* |  |
| Fig. S2 Melting curves of candidate reference genes and target gene in *S. superba*  Melting temperatures were visualized by plotting the negative first derivative of fluorescence relative to the temperature in Celsius [-(d/dT)]. | | |
