# Supplementary material for: Identification and Validation of Reference Genes for Gene Expression Analysis in Schima superba
Source: Genes (Basel). 2021 May 13;12(5):732. doi: 10.3390/genes12050732 (PMC8153319; doi:10.3390/genes12050732)
Supplement: Supplementary file 1 [file genes-12-00732-s001.zip › Additional file/Additional file 4 Table S2.docx]

>Ssu18G01981.1

ATGGCCGATGCTGAAGATATTCAGCCCCTTGTCTGTGACAATGGAACTGGAATGGTGAAGGCTGGGTTTGCTGGTGATGATGCCCCTAGGGCTGTTTTCCCCAGTATTGTTGGTCGACCCAGGCACACTGGTGTCATGGTTGGGATGGGCCAGAAGGATGCTTATGTAGGTGATGAAGCCCAATCCAAAAGAGGTATTCTTACCTTGAAGTATCCTATTGAACATGGTATTGTCAGCAACTGGGATGACATGGAAAAGATCTGGCATCACACATTCTACAATGAGCTCCGTGTTGCTCCTGAAGAGCACCCAGTGCTTCTTACAGAGGCACCACTCAACCCTAAGGCCAACAGAGAAAAGATGACACAAATTATGTTTGAGACTTTTAACGTTCCTGCCATGTATGTTGCCATCCAGGCTGTTCTTTCTCTATATGCCAGTGGTCGTACAACTGGTATTGTGCTGGATTCTGGTGATGGTGTGAGTCACACTGTGCCTATCTATGAGGGGTATGCTCTTCCTCATGCTATCCTCCGTCTTGACCTTGCTGGCCGTGATCTAACAGATGCCCTGATGAAGATCCTTACAGAAAGAGGTTACATGTTCACCACCACTGCTGAACGGGAAATTGTCCGTGACATGAAGGAAAAACTTGCATATGTCGCTCTTGACTTTGAGCAAGAGCTGGAGACTGCGAAAGAGCAGCTCCTCAGTCGAAAAGAACTATGA

>Ssu11G00151.1

ATGTTGAACTCGTCGCTGTTAACGATCCTTTCATCACCACTGATTACATGGGAGGAATCAGAGGGCAACCTAAAGGGGATATTGGGATTCACTGAAGATGATGTGGTCTCCTCTGACTTCGTGGGTGACAGCAGGTCAAGCATCTTTGATGCCAAGGCCGGGATTGCCTTGAATGACAACTTTGTGAAACTTGTCTCTTGGTATGACAATGAATGGGGTTACAGTGATTTTGTGAACTTGTTTATTTGTATGTTCAGTTGGAGGATCATCAAAGGTTCTGATAAGAAGATCAAGATCGGAATCAACGGCTTCGGAAGGATCGGCCGTCTCGTTGCAAGAGTTGCTCTCCAGAGAAACGATGTTGAACTCGTCGCTGTTAACGATCCTTTCATCACCACTGATTACATGTTGGATTTCTTGACAAGCGGTGTCATTAGAGTGCGTAGATGGGAGGAATCAGAGGGCAAACTAAAGGGGATATTGGGATTCACTGAAGAAGATGTGGTCTCCACTGACTTCGTGGGTGACAGCAGGTCAAGCATCTTTGATGCCAAAGCCGGGATTGCCTTGAATGACAACTTTGTGAAACTTGTCTCTTGGTATGACAATGAATGGGGTTACAGTTCACGCGTGATTGACTTGATCGTTCACATGGCTTGTGTTCAACCTTGA

>Ssu09G01555.1

ATGGTTTCTTTGGTGTTTGATTTTCTATTGAAAACTCAGATGGCTCGTACGAAGCAAACTGCTCGCAAGTCGACCGGAGGCAAGGCGCCAAGGAAGCAGCTTGCAACCAAGGCTGCTCGCAAGTCTGCCCCAACTACCGGAGGAGTGAAGAAGCCCCACAGATACAGACCTGGAACTGTTGCTCTTCGTGAAATTCGCAAGTACCAGAAGAGTACTGAACTCTTGATCAGGAAACTGCCATTCCAGAGGCTTGTTCGTGAGATTGCGCAGGACTTTAAGACTGACCTGAGGTTCCAGAGCCATGCAGTCTTGGCATTGCAGGAGGCAGCAGAGGCATATCTTGTTGGGTTGTTTGAAGATACTAATCTTTGTGCTATTCATGCCAAGCGGGTCACTATCATGCCTAAAGATATTCAGCTTGCAAGGAGAATTAGGGGTGAGAGAGCTTAG

>Ssu04G01886.1

ATGAGGGAGGTGTTAAGCATTCACATAGGACAAGCAGGAATTCAAGTGGGGAATTCATGCTGGGAGCTCTACTGTCTCGAGCATGGCATTCTTCCCGATGGCATGATGCCTAGTGACACCACAGTTGGTGTTGCACACGATGCCTTCAATACCTTCTTCAGCGAAACCAATGCTGGCAAGCATGTGCCTAGGGCAATATTTGTTGATCTGGAACCGACTGTGGTTGATGAAGTTAGGACTGGGACTTACAGACAGCTTTTTCATCCCGAGCAACTTATTTCTGGAAAAGAGGATGCTGCTAATAACTTTGCCAGAGGACATTATACAGTGGGAAAGGACATCATTGAAATATGCCTCGATCGTGTGAGAAAATTAGCTGACAATTGCACTGGCTTGCAAGGGTTTTTGGTAACTAACGCTGTTGGTGGTGGCACTGGTTCTGGATTGGGGTCCTTGCTTCTGGAGCGCTTGTCTGTAGAATATGGAAAGAAGTCAAAACTTGGCTTCACCATCTATCCTTCTCCTCAGGTCTCAACTGCAGTTGTGGAGCCTTACAACAGTGTGTTGTCCACTCATTCCCTACTTGAACACACAGATGTTGCCGTGCTCTTAGACAATGAAGCTATATATGATATCTGCAAGAGATCCTTAGACATTGAAAGGCCAACTTACACCAACTTAAATCGCTTGATATCTCAAGTCATCTCATCATTGACCACTTCCCTGAGGTTTGATGGAGCCATTAATGTGGACATTACAGAGTTCCAAACTAACCTTGTGCCATATCCTCGTATTCATTTCATGCTGTCGTCTTATGCACCGGTGATCTCAGCTGCAAAAGCATACCATGAGCAGCTATCTGTTCCTGAAATCACAAATTCTGTGTTTGAGCCCCCAAATATGATGGCCAAATGTGACCCAAGGCACGGAAAATACATGGCATGTTGTTTGATGTATCGTGGAGATGTTGTACCAAAGGATGTCAATGCTGCTGTTGGCACCATTAAGACTAAACGGACCGTTCAGTTTGTCGACTGGTGCCCAACTGGTTTCAAGTGTGGTATTAACTATCAGCCACCGACAGTGGTACCCGGGGGTGATCTTGCGAAGGTGCCAGCGTGCGGTTTGCATGATAAGCAACAACACAGCAGTGGCAGAGGTTTTCTCCCGAATTGA

>Ssu18G00414.1

ATGAGAGAGTGCATCTCGATCCACATCGGTCAGGCCGGTATCCAGGTCGGAAACGCCTGTTGGGAGCTTTACTGTCTTGAACATGGCATTCAGCCTGATGGACAAATGCCGAGCGACAAGACCGTCGGTGGAGGTGATGATGCCTTCAACACCTTCTTCAGCGAAACCGGCGCCGGAAAGCATGTCCCTCGCGCAGTTTTCGTAGATCTGGAACCTACCGTCATTGATGAGGTGAGGACCGGCACGTACCGCCAACTGTTCCACCCAGAGCAACTCATCAGCGGCAAAGAAGATGCCGCCAACAACTTTGCCAGAGGCCATTATACCATTGGGAAAGAAATTGTAGATCTGTGCCTGGATCGGATCAGGAAGCTTGCCGACAACTGCACGGGGCTCCAAGGTTTCCTGGTTTTCCATGCTGTGGGTGGTGGTACTGGATCTGGCCTTGGATCTCTGCTTCTTGAAAGGCTTTCCGTGGATTACGGAAAGAAATCGAAGCTTGGGTTCACTGTTTACCCATCGCCTCAGATCTCAACCTCTGTTGTTGAACCCTACAACAGTGTCTTGTCCACTCACTCTCTGCTAGAGCACACTGATGTTGCTGTACTCCTCGACAATGAGGCGATCTATGACATTTGCCGCAGATCCCTCGACATTGAGCGACCCACCTACACCAATCTCAACAGGCTTGTCTCTCAGGTCATTTCATCGCTGACTGCTTCTCTCCGTTTTGATGGAGCCCTTAATGTGGATGTGAACGAGTTCCAGACTAATTTGGTCCCATACCCAAGAATCCATTTCATGCTTTCTTCATACGCACCTGTTATCTCTGCTGAAAAGGCCTATCACGAGCAGCTCTCTGTTGCAGAAATCACAAACAGTGCTTTTGAACCATCATCTATGATGGTTAAATGTGATCCTCGCCATGGGAAGTACATGGCTTGCTGTTTGATGTACAGGGGTGATGTGGTGCCCAAGGATGTGAATGCTGCTGTGGCCACAATCAAGACCAAGAGGACCATTCAGTTTGTGGACTGGTGCCCAACTGGGTTCAAATGCGGTATCAACTACCAGCCACCCACCGTGGTGCCTGGCGGTGACTTGGCCAAGGTCCAGAGAGCTGTTTGCATGATCTCGAACTCAACCAGTGTTGCTGAGGTGTTCTCAAGGATCGATCACAAGTTTGATCTGATGTATGCCAAGCGTGCCTTTGTTCACTGGTATGTGGGTGAGGGTATGGAAGAGGGAGAGTTCTCCGAGGCTAGGGAGGACTTGGCTGCTCTGGAGAAGGATTATGAGGAAGTTGGTGCTGAGTCTGCTGAAGGCGAAGATGATGAGGGTGAGGAGTACTAA

>Ssu05G02026.1

ATGGCGGAGGACAAGTACAATCTGAAGAACCCGGCGGTGAAGAGGATTCTCCAGGAGGTCAAGGAGATGCAATCGAACCCTTCAGATGATTTCATGAGCCTCCCTCTTGAGGAGAATATATTTGAATGGCAATTTGCAATCAGGGGACCCCGTGATTCTGAGTTTGAGGGAGGCATCTATCATGGACGAATCCAATTGCCAGCAGAATACCCATTCAAGCCTCCCTCATTTATGCTGTTGACGCCAAATGGCCGTTTTGAAACACAGACTAAGATATGTCTAAGCATATCAAATCATCATCCTGAGCACTGGCAGCCATCATGGAGTGTTCGAACTGCTTTAGTTGCTCTAATTGCATTCATGCCCACCAACCCTAATGGTGCATTGGGCTCGTTAGACTACAAGAAAGAAGAAAGGCATGCTCTGGCAATTAAATCTCGTGAAGCTGCCCCAAGATTTGGTACTCCTGAACGGCAAAAACTAATCGATGAGATCCATGAATATATGCTAAGTAAGGCACCCCCGGTTCCTCAGCTCAATCCCTCGCAGGCTTCTGAAGAACATTCTACTGATAAAGAGAACGAAGCTAACCAGATTGTTCAAGATGCCGGTGCAGTAGCTGCTGGGGAAGGGATTACAAATCCAGCTGTGGGTGACAGGATTGTTGAAGAACCGCATGAAGCTCCTTTGAACGTGAATCCCAGACCCGAGGGTATGGGGGTATCTAGACAGGTTCATTCTGGACCCACAAGTGAGCAGCTCCTGCAGAGACCGGCACCAAGGGTTCAGAAACCGGCTGATGATCGCCTGTTCACATGGGCTGCTGTTGGACTTACCATTGCTATAGTTGTTCTGCTGTTGAAGAAGTTCATGAAAGCTAGTGGACATGGTGCCGTTTTCATGGATGGATCATAG

>Ssu11G00412.1

ATGTCGACTCCTGCAAGGAAGAGGCTGATGAGGGATTTTAAGAGGTTGCAGCAAGACCCTCCTGCAGGCATCAGTGGTGCTCCTCAAGATAACAATATAATGCTTTGGAATGCTGTTATATTTGGTCCTGATGACACCCCATGGGATGGAGGTACTTTTAAATTGACACTTCAATTTTCAGAGGATTATCCAAATAAACCACCAACAGTGCGCTTTGTTTCCCGAATGTTCCATCCAAATATTTATGCGGATGGAAGTATTTGTTTGGATATTCTACAAAATCAGTGGAGTCCTATCTATGATGTGGCTGCAATACTTACATCCATTCAGTCATTGCTTTGTGACCCAAACCCAAATTCCCCTGCCAATTCTGAAGCTGCACGGATGTTTAGCGAGAACAAGCGTGAGTACAACAGGAGAGTACGGGAGATCGTTGAGCAGAGTTGGACGGCTGACTGA

>Ssu14G00119.1

ATGGGATTTGGACTGCTTTTGATCTTCCCTGATGTACGCTTAGTTCCTTATGACTGTTTGACGGATGAGAGTTTCTTGCAAAATTTTCTTGGGAAGCTGCTTGAGTTGTTCTATTACTTTGTATTGTTTCGGCAGGTTGCGTTCAGGACAAAGGTTTTTCACCCTAACATCAACAGTAATGGCAGCATTTGTCTTGATATTTTGAAGGAACAGTGGAGCCCTGCACTTACCATTTCCAAGGTGTTGCTCTCCATCTGCTCTCTGTTAACAGACCCAAACCCTGATGACCCTTTGGTGCCGGAGATTGCTCACATGTACAAGACAGACCGGAGCAAGTATGAGACAACCGCCAGGAGCTGGACCCAGAAGTATGCCATGGGTTAA

>Ssu16G01394.1

ATGGCAGAGAAAGCATGTGTAAAGCGCCTTCAGAAGGAATATAGAGCCCTTTGCAAAGAACCTGTTTCTCATGTCACAGCCCGACCTTCCCCAAATGATATTCTCGAGTGGCATTATGTACTAGAAGGAAGTGAAGGAACACCTTTTGCGGGTGGATATTACTACGGGAAGATCAAGTTTCCTCCAGAGTATCCATTTAAACCTCCAGGAATCAGTATGACTACTCCAAATGGACGGTTTATGACGCAAAAGAAAATCTGCCTGTCTATGAGTGATTTTCATCCAGAAAGTTGGAATCCAATGTGGTCTGTATCAAGCATACTTACAGGCCTTCTCTCATTCATGATGGACAACAGTCCCACAACTGGCAGTGTAACTACAACTGTTGCTGAAAAGCAACGACTAGCAAAGTCTAGCCTTGCTTTCAATTGTAAGAACCCAACATTCAGGAAAATGTTCCCAGAGTATGTGGAGAAGTATGAACAGCAGCAGCTTTCTGAGCAGCCTGTTCCAGAGCAGGTGTCAAATGTCCCTACTCAACAAGGAGCTCACCGAATTTTATTGAAGAAAATTGAAAATAATTCCACGAAAGAGGACCAGAATAAGGTAGAAGAAGCCCCAAAGGATCTGAATAACCGCAGGAAGCAGTCTTTCCCAACCTGGATGCTGCTGTTACTGGTTTCCATCTTCGGTGTTGTAATGGCACTGCCACTGCTTCAGCTTTGA

>Ssu16G00399.1

ATGTCTCCATTAGTTTCAGACCTGCACCTTTATGATATAGCAAATGTCAAGGGAGTTGCTGCTGATCTCAGTCACTGCAATACTCCCTCTCAAGTTTTGGACTTCACAGGAGCTTCTGAGTTGGCCAATTGTCTGAAAGGTGTTAATGTGGTCGTCATCCCTGCTGGAGTTCCAAGGAAGCCGGGTATGACCCGCGATGACCTCTTCAACATCAATGCCAACATAGTGAAGACCTTGGTTGAGGCTGTTGCTGATAACTGCCCTGATGCCTTTATCCACATCATTAGCAATCCAGTTAACTCTACAGTGCCCATAGCAGCAGAAGTTTTGAAGCAGAAGGGTGTGTATGATCCAAAGAAGCTCTTTGGTGTCACTACTCTAGATGTCGTGAGAGCAAACACATTTGTTGCTCAGAAGAAGAACCTGAAGCTTATCGATGTTGATGTCCCAGTTGTGGGTGGACATGCCGGCATAACTATTCTACCCCTGCTGTCAAAGACAAAACCCTCTGTTAGTTTCACAGATGAAGAAGTACACGAGCTAACTGTGAGGATCCAAAATGCTGGGACGGAAGTCGTGGAGGCAAAGGCTGGTGCAGGGTCTGCTACCCTGTCAATGGCATATGCAGCTGCAAGATTTGTTGAGTCCTCTCTTCCGCGCTCTTGA

>Ssu06G01581.1

ATGTCTTGCTGCGGAGGAAACTGTGGATGCGGCTCTGGCTGCAGTTGCGGCAGCGGCTGCGGAGGATGCAAGATGTACCCTGACATGAGCTACTCTGAGAAGACCACCACTGAGACCCTTATTGTTGGTCTTGCCCCAAAGAAGACATACTTCGAGGGAACTGAGATGGGTGTGGCAGCTGAGAATGGTTGCAAGTGTGGAGCCAACTGCACCTGTGACCCATGCACCTGCAAATGA

>Ssu15G01370.1

ATGGCCGATCAGCTCACGGACGATCAGATCTCCGAATTTAAGGAGGCTTTCAGCCTCTTCGACAAGGACGGAGATGGTTGTATCACTACCAAGGAGCTTGGAACGGTGATGCGGTCATTGGGGCAGAACCCGACAGAAGCTGAGCTCCAGGACATGATTAATGAGGTTGATGCTGATGGAAATGGGACAATTGATTTCCCAGAGTTCCTCAACCTGATGGCCCGGAAAATGAAAGATACAGACTCCGAGGAGGAGCTCAAGGAAGCTTTCCGGGTTTTCGACAAGGACCAGAATGGGTTCATTTCTGCAGCTGAGCTCCGCCACGTTATGACGAATCTTGGTGAGAAGCTCACAGATGAGGAAGTCGACGAGATGATTCGTGAGGCTGATGTGGACGGTGATGGGCAGATCAACTACGAGGAGTTCGTGAAGGTCATGATGGCGAAGTGA

>Ssu13G00009.1

ATGCTAGCCCATCTGGCCATGCTGATGAAGCATTATGGAGCTCTTAGCAATGCAATTCAGATGATAATTGCTGAAACAGATGGTACTGTTAATAACCAGGAGCTTGTAATGAGGGCCAGGGAATTGCTGAAAAGCCAGGGTTTCACTCAGCGTCCAGGCCTCTATTGCAGTGACCATCATGTTGACACTCCTTTTGTGTGCTGA

>Ssu07G01240.1

ATGTCGGATGAGGAGCATCACTTCGAGTCGAAGGCCGACGCAGGTGCCTCCAAGACCTATCCACAGCAGGCTGGAACAATCCGCAAGAACGGCTACATAGTCATCAAGAACAGGCCTTGCAAGGTTGTGGAGGTCTCCACCTCAAAAACAGGCAAGCACGGACATGCAAAGTGCCACTTTGTTGCAATTGATATCTTTAATGGCAAGAAGCTTGAAGATATTGTCCCTTCCTCCCACAATTGTGATGTTCCCCATGTTAATCGTACTGACTACCAGCTGATTGATATCTCCGAAGATGGTTTTGTGAGTCTTTTGACTGAAAATGGAAATACCAAGGATGATTTGAGGCTCCCCACTGATGACTCTCTGCTCACCCAGATTAAGGATGGGTTTGGTGAAGGAAAGGACCTTGTTGTGACTGTCATGTCTGCAATGGGAGAAGAACAAATCTGCGCCCTAAAGGACATTGGTCCTAAAAATTAA

>Ssu11G01342.1

ATGTTTTTGTTCGATTGGTTCTATGGAATTCTCGCATCACTCGGTCTGTGGCAGAAGGAGGCTAAGATCTTGTTTTTGGGGCTTGATAATGCTGGCAAGACGACGTTACTTCATATGTTGAAAGATGAGAGATTGGTTCAGCATCAGCCAACCCAATACCCAACATCGGAGGAGCTTAGTATCGGGCAAATAAAGTTTAAGGCTTTTGATTTGGGTGGCCATCAGATTGCCCGCAGAGTTTGGAAAGATTACTATGCCAAGGTGGATGCTGTTGTGTACTTAGTGGATGCTTATGACAAGGAGAGATTTGCCGAGTCGAAAAAGGAGCTGGATGCTCTCCTGTCTGATGAGTCCTTGGCTACTGTTCCCTTCCTAATTTTGGGCAACAAGATTGATATCCCTTATGCTGCTTCGGAAGATGAGTTGCGTTACCACATGGGTCTGACCGGCATCACCACAGGGAAGGGGAAGGTAAACCTGGCAGACTCAGCTGTCCGTCCACTCGAGGTGTTCATGTGCAGTATTGTCCGCAAAATGGGCTACGGGGATGGCTTCAAGTGGGTTTCTCAGTACATCAAGTAG

>Ssu08G00694.1

ATGGCTGTTCCTTTGATCACCAAGAAAATTGTGAAGAAGCGTGTGAAGAAGTTCAAGAGGACCCAAAGCGACTGGAAGATCTCTGTGAAGCAAAGCTGGCGCAGGCCCAAGGGTATTGATTCCCGCGTGAGGAGGAAGTTCAAAGGATGTGTTCTCATGCCTAACATTGGTTATGGTTCAGACAAGAAGACTCGCCATTATCTTCCTAATGGGTTTAAGAAATTCCTTGTCCACAATGTCAAAGAGCTGGAAGTTTTGATGATGCACAACAGGACTTACTGTGCGGAAATAGCACACAATGTATCGACTCAGAAACGGAAGGAGATTGTTGAGCGTGCAGCACAGCTGGATATTGTTGTTACCAACAAATTGGCAAGGTTGCGCAGCCAGGAAGATGAATGA

>Ssu03G00328.1

ATGCGAGAAATCCTCCACATCCAGGGCGGCCAATGTGGCAACCAGATCGGGGCCAAGTTCTGGGAGGTGATCTGTGACGAGCACGGAATCGATTACTCTGGAAAGTACAGTGGCGACTCCGACCTCCAGCTCGAGCGAATCAACGTCTATTACAATGAAGCCAGCGGTGGAAGGTACGTCCCTCGCGCCGTCCTCATGGACCTCGAGCCAGGCACCATGGATTCTGTCCGATCTGGTCCTTTCGGGCAGATCTTCCGACCGGACAATTTCGTTTTCGGCCAGTCAGGTGCCGGAAACAATTGGGCTAAAGGCCATTACACCGAAGGAGCTGAGCTCATCGATTCCGTCCTCGATGTTGTGAGGAAGGAGGCTGAGAATTGTGATTGTTTGCAGGGATTTCAAGTTTGTCATTCTTTGGGTGGAGGCACTGGATCTGGTATGGGCACCCTTCTCATTTCCAAGATCAGGGAGGAGTATCCAGATCGCATGATGTTGACATTTTCAGTCTTTCCTTCTCCCAAAGTATCTGATACTGTTGTTGAGCCATACAATGCTACCCTTTCTGTTCATCAACTTGTTGAGAACGCTGATGAATGTATGGTTTTGGACAATGAAGCTCTCTACGACATCTGCTTCCGAACTCTCAAGCTTGCAACCCCTACTTTTGGCGATCTCAACCACCTGATCTCTGCTACCATGAGCGGTGTCACATGCTGTCTTCGGTTCCCTGGACAGCTTAACTCCGACCTTCGGAAACTTGCAGTTAACCTTATTCCATTCCCTCGTCTCCACTTCTTCATGGTCGGGTTTGCACCCCTAACCTCCAGAGGCTCCCAGCAGTACCGAGCGCTCACCGTCCCTGAACTGACCCAACAGATGTGGGATGCCAAGAACATGATGTGTGCTGCCGACCCACGTCACGGCCGCTACTTAACTGCTTCAGCCATGTTCCGTGGTAAGATGAGCACTAAAGAAGTCGATGAACAGATGATAAATGTCCAGAACAAGAACTCCTCATACTTTGTTGAGTGGATACCCAACAATGTCAAGTCTAGTGTCTGTGACATCCCCCCCAAGGGTCTGAAAATGGCATCGACTTTCATTGGGAATTCAACTTCAATTCAAGAGATGTTTAGGCGTGTGAGCGAGCAATTCACTGCCATGTTTAGGCGGAAGGCCTTCTTGCATTGGTACACTGGTGAGGGAATGGACGAGATGGAGTTTACCGAGGCTGAGAGTAACATGAATGATCTTGTGGCCGAGTACCAGCAATACCAGGACGCAACTGCGGATGATGAGGAATACGAGGATGAAGAAGAGGAGGTTGCTGCTTGA

>Ssu09G01228.1

ATGGAGGAGGGTCGTTTGTGTCAGTGGAGCGTGATTCGATCTATTCTCGCCATTATTCAATGGTGGGGCTTCAATGTTACCGTAATCATCATGAACAAATGGATCTTCCAGGTGCTAAAGCTTAAGCCACTCATAGTGGTTGATCCTGAAGATCGATGGAGAAGGATCTTTCCCATGTCTTTTGTCTTTTGTATCAACATAGTATTGGGGAATGTAAGCTTGCGTTACATTCCTGTTTCTTTTATGCAAACCATAAAGTCGTTTACCCCTGCAACAACAGTTATTTTGCAGTGGTTAGTTTGGAGGAAAAGCTTCGATTCACGAATTTGGGCTTCACTGGTGCCCATTGTAGGAGGAATTCTTCTAACTTCTATTACTGAGCTTAGTTTCAATATGTTTGGATTTTGTGCTGCCTTGTTTGGTTGTCTTGCTACTTCTACAAAGACTATCCTTGCAGAGTCTCTGCTGCATGGCTACAAGTTTGACAGTTATATCAATCATCTCATTGGACATGCATTATATGCATGTGGAAAGCTGTGGAGTCGACCAAATAGCTTTGGCAAAAACATTGTTATTCAAATAAAAAGATTCGCTGGATATTATGATGGAAAGCTTCATTAG

>Ssu06G00017.1

ATGCCAGAAAAAACCATGAGCATATCTGTAAATGGGCAATCTCAGGTCCCTCCCGGCTTCCGGTTTCATCCAACAGAAGAGGAGCTCTTGCAATACTACCTGAGGAAGAAGGTTTCATTCGAGAAGATTGACTTGGATGTCATTCGCGATGTCGATCTCAACAAACTCGAGCCTTGGGATATACAAGAGAAGTGTAGAATAGGATCGACTCCACAGAATGATTGGTACTTCTTCAGCCACAAAGACAAGAAGTACCCAACTGGTACGAGAACAAATCGAGCCACTGCTGCCGGGTTTTGGAAGGCTACAGGCCGTGATAAGGTGATCTACAGCAACTCCAAACGGATTGGGATGAGGAAGACGCTGGTTTTCTACAAAGGTCGGGCCCCTCACGGCCAAAAGTCCGATTGGATCATGCATGAGTATAGACTGGATGACAATTCCTGTGATACCAATAATGTTTCCGATGCTACTGGAGAAGCAACACAAGAAGAGGGATGGGTGGTTTGTCGTATCTTCAAGAAGAAAAACCACCACAAAACCCTCCTAGACAGCAACAACAACAACAACATCAACAACATCAACTGCCCTTTCAATTCAGCTCTCAATCTCGACACAAGAACCCAAATACCTTCAAGCAGCGACGAAGGCACCTTAGAACAAATCCTTCACTACTTGGGCAGGACTTGCAAGAAGGAAGATCATGAAACAACAATCAACAACAGCCACGGATTTCTCATGCCCATCGACACGGCCCCAAAAAATAGCCTCCATGACAGGTTCATGAAGCTTCCAAGTCTAGAGAGCCCAAACTCCATCAGTAGCCAACCTTATCACCAACAAATTCAACTACATAATCTACAGATGCTCACAGACAACGAAGAAACAGGCCTTCATGACTGGACAGCCCTCGATCGGCTTGTGGCTTCACACCTCAATGGCCAAACTGAGACTTCAAAGCAGATAACATCATGCTTTAACGATCCCAACATGGGTTTTGGTTCCCTTCCCTATCATGATCTGCAACTACAGCAGCAGCAGCAACAACTACGATCATCGTCATCATCACCGTCCAATAGACCACCTTTCCATGGCACGCCTACCCAGGATGATAATGGGGAATTTGACCTTTGGAGCTTCGCGCGATCTTCCTTGTCCAACACTTCTGTATAA
